# Biomarkers of aging associated with past treatments in breast cancer survivors

**DOI:** 10.1038/s41523-017-0050-6

**Published:** 2017-12-12

**Authors:** Zorica Scuric, Judith E. Carroll, Julienne E. Bower, Sam Ramos-Perlberg, Laura Petersen, Stephanie Esquivel, Matt Hogan, Aaron M. Chapman, Michael R. Irwin, Elizabeth C. Breen, Patricia A. Ganz, Robert Schiestl

**Affiliations:** 1 UCLA Fielding School of Public Health, Los Angeles, CA USA; 2 Department of Psychiatry & Biobehavioral Sciences, UCLA David Geffen School of Medicine, Los Angeles, CA USA; 3 Cousins Center for Psychoneuroimmunology, UCLA Semel Institute for Neuroscience and Human Behavior, Mail Code 707624, 300 Medical Plaza, Suite 3330, Los Angeles, CA 90095-7076 USA; 4 UCLA Jonsson Comprehensive Cancer Center, 650 Charles Young Drive South, Room A2-125 CHS, Los Angeles, CA 90095-6900 USA; 5 UCLA Department of Psychology, Los Angeles, CA USA; 6 UCLA Departments of Pathology, Environmental Health, and Radiation Oncology, and UCLA School of Public Health, Los Angeles, CA USA

## Abstract

Radiation and chemotherapy are effective treatments for cancer, but are also toxic to healthy cells. Little is known about whether prior exposure to these treatments is related to markers of cellular aging years later in breast cancer survivors. We examined whether past exposure to chemotherapy and/or radiation treatment was associated with DNA damage, telomerase activity, and telomere length 3–6 years after completion of primary treatments in breast cancer survivors (stage 0–IIIA breast cancer at diagnosis). We also examined the relationship of these cellular aging markers with plasma levels of Interleukin (IL)-6, soluble TNF-receptor-II (sTNF-RII), and C-reactive protein (CRP). Ninety-four women (36.4–69.5 years; 80% white) were evaluated. Analyses adjusting for age, race, BMI, and years from last treatment found that women who had prior exposure to chemotherapy and/or radiation compared to women who had previously received surgery alone were more likely to have higher levels of DNA damage (*P *= .02) and lower telomerase activity (*P* = .02), but did not have differences in telomere length. More DNA damage and lower telomerase were each associated with higher levels of sTNF-RII (*P*’s < .05). We found that exposure to chemotherapy and/or radiation 3–6 years prior was associated with markers of cellular aging, including higher DNA damage and lower telomerase activity, in post-treatment breast cancer survivors. Furthermore, these measures were associated with elevated inflammatory activation, as indexed by sTNF-RII. Given that these differences were observed many years after the treatment, the findings suggest a long lasting effect of chemotherapy and/or radiation exposure.

## Therapeutics: past chemotherapy and radiation linked to cellular aging

Past exposure to chemotherapy and radiation is associated with signs of cellular aging among breast cancer survivors. Zorica Scuric, Judith Carroll, and colleagues from the University of California, Los Angeles, USA, tested markers of biological aging and inflammation in blood samples taken from women 3–6 years after their initial treatment for stage 0–IIIA breast cancer. They found that women who had received chemotherapy and/or radiation were more likely to have high levels of DNA damage, lower activity of telomerase—an enzyme involved in maintaining the length of chromosomes—and elevated inflammatory activation compared to women who underwent surgery alone. The findings point to an enduring biological effect of chemotherapy and radiation, and suggest that some breast cancer survivors may be vulnerable to accelerated aging because of their prior treatment.

## Introduction

Successful treatment of cancer has dramatically increased the number of cancer survivors, largely through the application of multi-modal therapies, including surgery, radiation, chemotherapy, and biotherapy.^1–3^ However, the potential for long-term detrimental impact of these treatments and their consequences to health and quality of life for survivors is of increasing concern.^4,5^ Indeed, cancer survivors are at increased risk for earlier disability, chronic disease, and death, raising the possibility that some cancer treatments may accelerate the aging process.^2,5–12^ Among childhood cancer survivors in particular, the accumulated burden of chronic conditions has been attributed to the exposure to these lifesaving treatments and their late effects, often leading to premature death in comparison to unaffected sibling controls.^7^ Less is known about the potential for treatment-associated increased morbidity and acceleration in aging among adult cancer survivors, although as more long-term studies are done in adult survivors, similar concerns have been advanced.^5,12–14^

Radiation and many chemotherapeutic agents are cytotoxic, resulting in damage to cancer cells and the ultimate death of these cells when treatment is successful. Non-cancer cells are also affected by these same treatments, resulting in death of cells in normal tissues. When a significant amount of damage accumulates within a cell from either radiation or cytotoxic chemotherapy, the cell will either initiate apoptosis leading to cell death, or growth arrest to enable the cell to repair the damage. When the damage exceeds internal capacity to repair, the growth arrest becomes permanent, a state referred to as cellular senescence. Senescent cells are considered a key player in the aging process.^15,16^ Senescent cells are sources of inflammation, releasing numerous secretory factors, including cytokines, chemokines, and various damage-associated molecular patterns that propagate and promote inflammatory activity from other cells and in nearby tissue microenvironments.^17–19^ This milieu of inflammatory secretory factors further promotes aging processes and has been implicated in increased vulnerability to cancer, cardiovascular disease, diabetes, dementia, frailty, arthritis, and numerous other diseases of aging.^20^ Thus, diseases and conditions seen in advancing age develop as a consequence of cellular aging pathways, including senescence.

Recent animal models have characterized the impact of chemotherapy on the accumulation of senescent cells,^21^ and clinical studies have documented elevated levels of DNA damage^22^ and increased expression of *p16* ^*INK4a*^, a marker of senescence, within T cells of patients after chemotherapy.^23^ In parallel, studies have shown an increase in inflammatory markers during radiation and/or chemotherapy^24–26^ that may persist long after treatment completion.^12,27^ Additional markers of the biological aging process that may be relevant in the context of cancer treatments include telomere length, a repeat sequence of DNA at the end or chromosomes, which shortens with cellular replication and can initiate senescence, and telomerase activity, an enzyme that rebuilds telomeres,^28^ but whose function is no longer necessary in senescent cells. While some evidence suggests cancer treatments do not impact leukocyte telomere length,^23,29^ others have reported shorter telomere length among cancer survivors.^30–32^ Thus, whether cancer treatment impacts telomere length is inconclusive. On the other hand, whether cancer treatment impacts peripheral blood telomerase activity has not been tested. In the current study we examine the hypothesis that both chemotherapy and radiation will be associated with markers of biological aging, including higher levels of white blood cell (WBC) DNA damage, lower peripheral blood mononuclear cell (PBMC) telomerase activity, and shorter PBMC telomere length, that are all hallmarks of aged cells.^15^ We test this hypothesis in a well-characterized cohort of breast cancer survivors from the mind body study (MBS).^25,33^ In addition, given the link between cellular aging processes and inflammation, we also predicted that these cellular aging biomarkers would be related to proinflammatory secretory factors in breast cancer patients years after treatment completion.

## Results

### Participant characteristics

Ninety-four women were assessed at the MBS final visit (TF), 3–6 years after the initial diagnosis of breast cancer. Blood samples of satisfactory quantity and quality for analyses were available for total WBC DNA damage (*N* = 94), PBMC telomerase activity (*N* = 84; telomerase product generated (TPG) per 10,000 cells *M*[SD] = 22.7[19.4]), PBMC telomere length (*N* = 87; *M*[SD] = .72[.27]), and plasma cytokines (*N* = 94; IL-6 pg/mL *M*[SD] = 1.3[.99]; sTNF-RII, pg/mL *M*[SD] = 2228.4; C-reactive protein (CRP), mg/L *M*[SD] = 2.4[2.7]). Descriptive information on the complete MBS cohort has been reported elsewhere,^25,33^ and those who participated in the TF visit are representative of the full sample. Table 1 presents the descriptive data for these women overall, and stratified according to surgery or chemotherapy and/or radiation exposure (our primary case-control analysis). Additional supplementary tables report descriptive information for patients by various treatment subtypes (e.g., radiation alone, chemotherapy alone, and both radiation and chemotherapy combined; Supplement Table 1). In chemotherapy and/or radiation-exposed group compared to surgery alone, the demographic characteristics were similar; however, the surgery alone group had lower stage disease, were more likely to have mastectomies, and had lower rates of endocrine therapy, as would be expected.

**Table 1:** Participant medical and demographic characteristics

	Total sample	Surgery alone	Chemo and/or radiation	*P-*value
	*N* = 94	*N* = 15	*N* = 79	
Age, mean (SD)	56.5 (8.1)	58.1 (5.4)	56.2 (8.5)	.39
Body mass index (BMI), mean (SD)	25.7 (5.1)	26.0 (5.6)	25.6 (5.0)	.79
Years since diagnosis, mean (SD)	4.8 (0.7)	4.7 (.60)	4.8 (.68)	.50
Years since last treatment, mean (SD)	4.4 (0.6)	4.6 (.57)	4.4 (.65)	.16
Race, % White	80%	86.7%	78.5%	.47
Marital status, % Married	62.8%	53.3%	64.6%	.41
Education				.82
Post college	50%	53.3%	49.4%	
College	31%	33.3%	30.4%	
No college degree	19%	13.3%	20.3%	
Employment status				.08
Full or part-time	73%	86.7%	63.3%	
Not employed	27%	13.3%	36.7%	
Annual household income				.54
≥$100,000	59.6%	66.7%	58.2%	
<$100,000	40.4%	33.3%	41.8%	
Post-menopausal	80.9%	53%	51%	.93
Past hormone therapy (HT)	33.3%	28.6%	34.2%	.68
Surgery				.000
Mastectomy	34%	80%	25.3%	
Lumpectomy	66%	20%	74.7%	
Stage at diagnosis				.000
Stage 0	16%	53.3%	8.9%	
Stage I	46%	46.7%	45.6%	
Stage II	31%	0%	36.7%	
Stage III	7%	0%	8.9%	
Endocrine therapy	72%	40%	78.5%	.002
Had chemotherapy	54.2%	0%	64.6%	
Had radiation	72%	0%	86.1%	

Initial analyses examined whether demographic factors were related to our biological outcomes of interest. Age and longer time since treatment were associated with higher DNA damage (*P* < .002; *P* = .002). Other demographics were unrelated to DNA damage. We also tested for a possible role of current comorbidities as they might relate to our biological markers, including presence of allergies, arthritis, asthma, diabetes, glaucoma, heart disease, and hypertension, and found no statistical differences in frequency of these conditions by DNA damage group, and no significant mean differences in PBMC telomere length or WBC telomerase activity, with the exception of significantly lower telomerase activity in those with allergies (*P* = .02). With respect to PBMC telomerase activity, declines were observed with increasing age (*r* = −.23, *P* = .04), and shorter PBMC telomere length was associated with high BMI (*r* = −.21,* P* < .05). No other demographic factors related to these biomarkers. Subsequent models adjusted for age, race, BMI, and years since last treatment.

### Chemotherapy and/or radiation exposure

**Fig. 1:**
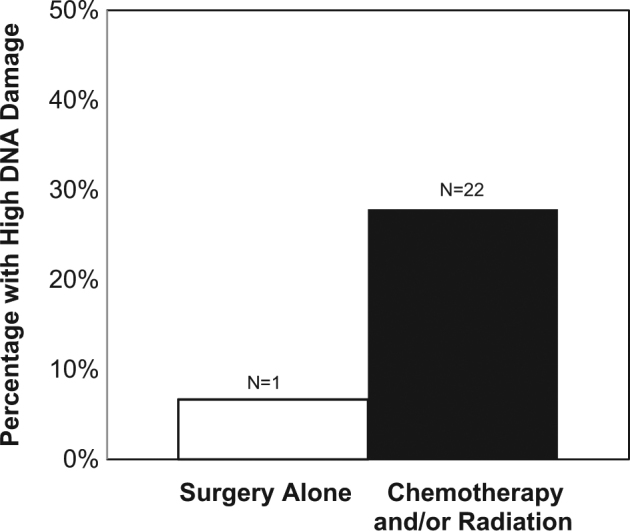
Percentage of breast cancer survivors with high DNA damage (top quartile) with either past exposure to chemotherapy and/or radiation or surgery alone. OR(95% CI) = 18.49(1.56, 218.9), *P* = .02

**Fig. 2:**
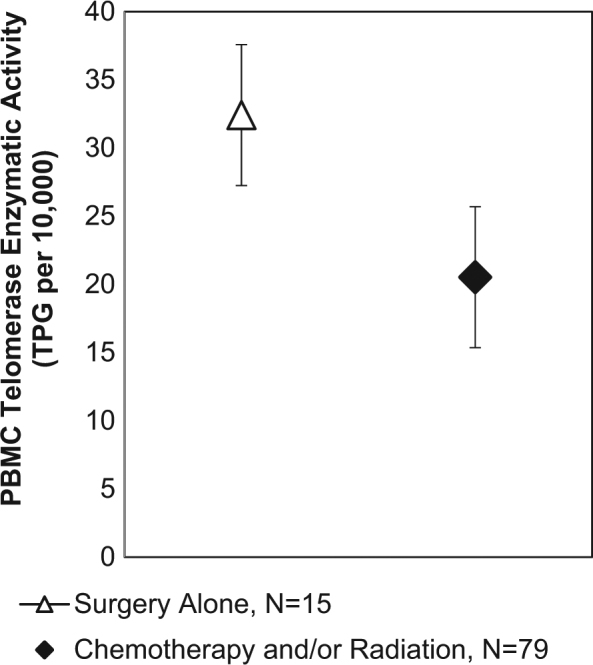
Estimated marginal mean ± SE of PBMC telomerase enzymatic activity (TPG per 10,000 cells) in breast cancer survivors with either past treatment exposure to chemotherapy and/or radiation or surgery alone, adjusting for age, race, BMI, and years from treatment exposure. B(SE) = −1.93(.83), *P* = .02. *Note*: Figure displays mean scores from non-transformed data, while statistical analyses use transformed telomerase activity (deciles) due to non-normal distribution in TPG values

To test our primary hypothesis that the overall effect of prior chemotherapy and radiation exposures would relate to markers of biological aging, we examined the effects of chemotherapy and/or radiation exposure on markers of cellular aging at TF (see Table 2). In adjusted models controlling for age, race, BMI, and years since last treatment, women who had been exposed to chemotherapy and/or radiation (i.e., any therapy in addition to surgery) were significantly more likely to have high levels of DNA damage compared to women who received surgery alone (*P* = .02; see Fig. 1). In addition, women exposed to chemotherapy and/or radiation had significantly lower telomerase activity compared to surgery alone (*P* = .02), and further adjustment for comorbid allergies did not modify this effect. Figure 2 presents the mean and standard error (SE) for telomerase enzymatic activity separated by exposure. Additional adjustment for endocrine therapy did not alter these effects (*P*’s remain <.05). The effect of chemotherapy and/or radiation exposure was associated with slightly higher mean values of sTNF-RII (2272 vs. 2038 pg/mL, *P* = .15), although not statistically significant. There were no differences in PBMC telomere length by exposure group. The Supplementary File and Supplemental Table 3 further explore the effect of radiation, chemotherapy, the combined effect of receiving both, and the type of chemotherapeutic agent on these aging markers.

**Table 2:** Multivariate analyses examining treatment exposure type predicting biomarkers of aging parameters at final visit (TF) adjusting for age, race, BMI, and years from treatment

	WBC DNA damage (high damage vs. low)			PBMC telomerase (deciles)			PBMC telomere length (T/S)		
Predictor	*β*(SE)^a^	OR(95% CI)^b^	*P*-value	*β*(SE)	Beta^c^	*P*-value	*β*(SE)	Beta	*P*-value
Covariates in the model									
Age (years)	0.15(.05)	1.16(1.06, 1.28)	.002	−0.09(.04)	−0.23	.04	−0.004(.004)	−0.11	.32
Race (White = 1)	1.14(1.23)	3.14(0.35, 28.59)	.31	0.05(.91)	0.007	.96	−0.11(.08)	−0.17	.16
Body mass index (kg/m^2^)	0.007(.06)	1.01(0.89, 1.14)	.92	−0.04(.07)	−0.07	.54	−0.01(.006)	−0.23	.04
Years from treatment	1.49(.47)	4.44(1.77, 11.16)	.002	−0.17(.48)	−0.04	.73	−0.05(.05)	−0.11	.32
Treatment exposure									
Chemotherapy and/or radiation^d^	2.92(1.26)	18.49(1.56, 218.9)	.02	−1.93(.83)	−0.25	.02	0.07(.08)	0.09	.39

^a^ Standard coefficient (*β*) and standard error (SE)

^b^ Odds ratio (OR) and 95% confidence interval (95% CI)

^c^ Unstandardized regression coefficient (Beta)

^d^ Compared to those receiving neither treatment, a surgery alone group (*N* = 15)

### Inflammation and biomarkers of aging

**Fig. 3:**
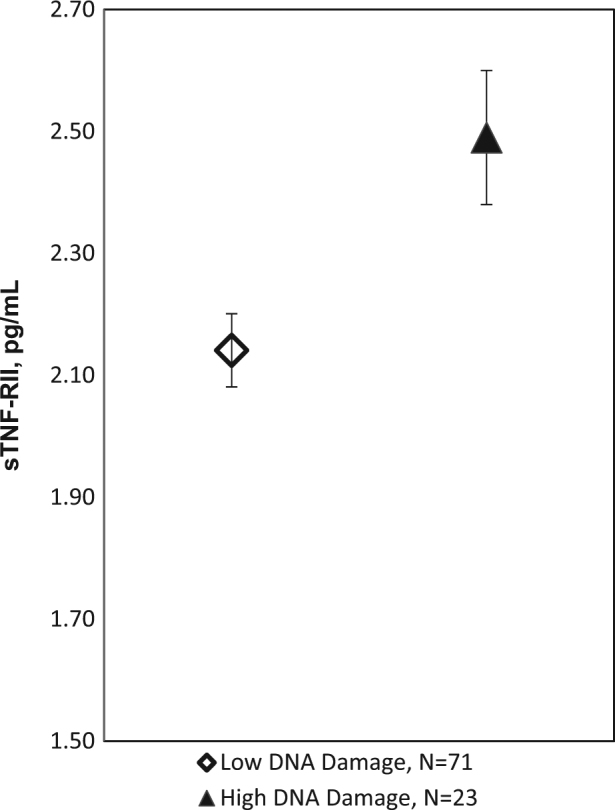
Mean ± SE of sTNF-RII within higher (top quartile) and lower (three lower quartiles) DNA damage groups adjusting for age, race, BMI, and years from treatment exposure. B(SE) = 349.8(127.5), *P* = .007

To determine the relationship of circulating markers of inflammation with biological aging indices we ran additional regression analyses. In adjusted models, sTNF-RII was related to DNA damage, such that women with high DNA damage exhibited significantly higher levels of sTNF-RII, *B*(SE) = 349.8(127.5), *P* = .007 (see Fig. 3), and modestly higher, but not significant, IL-6, *B*(SE) = .44(.24), *P* = .08. No associations were observed between DNA damage and CRP (*P* = .73). Telomerase activity was also associated with sTNF-RII levels, with lower telomerase activity related to marginally higher levels of sTNF-RII, *B*(SE) = −36.34(19.2), *P* = .06. Telomerase was unrelated to IL-6 (*P* = .90) and CRP (*P* = .57). Telomere length was unrelated to any of the inflammatory markers (*P*’s > .35).

## Discussion

Among the breast cancer survivors in the present analyses, we report significantly greater DNA damage and lower levels of telomerase enzymatic activity among women who had been exposed to chemotherapy and/or radiation therapy, compared to those who had not received either of these treatments. These differences were detectable even though exposure had occurred 3–6 years prior to the current study visit. Specifically, having been exposed to chemotherapy and/or radiation was associated with the highest levels of DNA damage and lower telomerase activity, both markers of greater cellular aging. Increased DNA damage has been reported in breast cancer survivors compared to healthy controls, although treatment-specific effects were not reported.^22^ In our analyses, greater DNA damage and lower telomerase activity were also associated with elevations in inflammatory activity, as indicated by sTNF-RII levels. sTNF-RII is a downstream marker of activity of the pro-inflammatory cytokine TNF-alpha, which is shed upon upregulation of inflammation via the TNF pathway.^34,35^ Elevated levels of sTNF-RII indicate activation of inflammation, with further implication for aging given the role of TNF-alpha in the proinflammatory secretory profile of senescent cells.^17–19^ Our previous work has demonstrated that sTNF-RII was the only elevated inflammatory marker during the first 6 months after finishing chemotherapy.^33^ The current findings suggest that exposure to cancer treatments that include chemotherapy and/or radiation are associated with longer-term increases in DNA damage and reductions in telomerase activity, indicators of biological aging that coincide with higher levels of inflammatory activation, including sTNF-RII in the present sample.

We did not observe differences in telomere length related to treatment exposure. However, we did see a significant relationship of PBMC TL with BMI, consistent with an independent association of adiposity with greater replicative cellular aging as indexed by shortened TL.^36^ These results are similar to other reports suggesting exposure to chemotherapy and radiation treatments may not consistently be related to blood cell telomere length shortening per se, but rather may drive aging via induction of DNA damage and cell senescence.^23,29–32^ Indeed, telomere length shortening occurs by cell replication, a process often halted during cancer treatments. Cellular senescence, on the other hand, is reached through either replicative exhaustion or cell stress pathways (i.e., DNA damage). Cellular aging among breast cancer patients may therefore occur independent of the telomere ends, and be driven more so by DNA damage.^15,16^ Our results contribute to a growing literature that proposes certain cancer treatment exposures may leave a lasting imprint on cellular biology by linking it to DNA damage, a pathway to cellular senescence.

Previous studies have linked effects of breast cancer treatment (chemotherapy and/or radiation) to higher levels of inflammatory markers,^12,24–26,33^ and separately, to elevated levels of a marker of cellular senescence, *p16* ^*INK4a*^.^23^ The current study adds to this research by directly capturing cell level DNA damage and telomerase enzymatic activity, and examining treatment-specific exposures and associations with pro-inflammatory cytokine levels measured from the same blood draw. Further research may benefit from the inclusion of a broader panel of markers of systemic inflammatory activity that are known to be released from senescent cells and a better characterization of DNA damage in treatment-exposed cancer survivors using markers like hOGG1 to discriminate between oxidative and non-oxidative damage.^37^ Host factors may also play an important role in determining the extent and duration of the damage seen in treatment-exposed cancer patients and associated inflammation, and further examination of patient-specific variability, such as genetic variants (e.g., hOGG1 variant S326C-OGG1^38^) that influence DNA repair capacity is warranted. Similarly, telomere length has also been found to be partially determined by genetic factors and inheritance,^39,40^ and future work should consider the role this might play.

Limitations exist in the current analyses. The cross-sectional nature of the study limits causal attribution. For example, although inflammatory cytokines are released from aged cells, this is not the only source, and the directionality of these relationships remains uncertain. Inflammation is also a recognized cause of aging,^41^ and further work should disentangle this in the context of cancer treatment exposure effects.^20^ Along this line, it is also possible that the treatments themselves are not directly responsible for increased DNA damage. Rather the biology of the cancer may dictate treatment regimen that contributes to lasting differences in DNA damage, although animal models do not support this conclusion. It is also possible that unmeasured differences exist between those receiving surgery alone compared to chemotherapy or radiation treatments that explains the differences in biomarkers of aging we report. Although, as can be seen in Table 1, the surgery only group appears to be similar to the other treatment group on the majority of demographic and medical factors, with the exception of being earlier stage, type of surgery received, and whether treated with endocrine therapy. Further adjustment by endocrine therapy in our models did not alter our results. Further research that assesses DNA damage using a within patient design, examining DNA damage prior to treatment exposure and years later, may help answer this remaining possibility. Likewise, future animal research could more carefully test this hypothesis by employing well-designed experimental models of cancer treatment exposures (e.g., radiation/chemotherapy), and observe pre-to-post DNA damage, telomerase activity, cellular senescence, and inflammatory activity from multiple cell sources. Moreover, our measure of DNA damage was in whole blood, while telomerase was assessed in purified mononuclear cells. DNA damage from treatment is most likely to be retained years later in the PBMC pool, rather than in all leukocytes. Neutrophils (predominant cell in whole blood leukocytes are not present in PBMCs) are short lived in circulation (e.g., weeks), while many of the other cells found in PBMCs remain in circulation for years. Future studies may want to consider whether examining DNA damage in PBMCs, rather than all leukocytes, may yield even more meaningful and relevant information regarding the lasting impact of treatment exposures on DNA damage. Further research is warranted in cancer survivors that provide a stronger link between these elevated markers of aging with subsequent risk for, or exacerbation of, existing comorbid conditions associated with aging. The current study has several strengths that should be noted. First, the study examines a well-characterized cohort of breast cancer survivors with medically verified reports of cancer treatment exposure who were then followed for several years after treatment. Second, the measurement of cellular DNA damage and telomerase activity, and linking this to inflammation in breast cancer survivors is novel and represents a unique and promising direction for future research.

## Conclusion

In conclusion, we followed breast cancer patients for 3–6 years after completion of primary treatment and found that those who had been exposed to chemotherapy and/or radiation exhibited elevated levels of DNA damage in peripheral blood cells and lower telomerase enzymatic activity. Both high DNA damage and low telomerase activity were associated with elevated levels of sTNF-RII, a biomarker of proinflammatory activation. These results are consistent with existing evidence that exposure to certain cancer treatments have a lasting impact on biological aging pathways, in particular the accumulation of senescent cells, and further suggest that elevated damage to DNA may be mechanisms through which senescent cells are formed in cancer patients. These findings support the hypothesis that cancer survivors may be vulnerable to accelerated aging due to the lasting effects of chemotherapy and radiation exposure.

## Methods

### Participants

Participants in the current study were from the UCLA MBS, a longitudinal, prospective cohort study of 190 women with early stage breast cancer enrolled after the end of primary breast cancer treatment, and prior to initiating adjuvant endocrine therapy, if indicated.^25,33,42–44^ All procedures were approved by the University of California, Los Angeles, Institutional Review Board, and all participants provided informed consent. The MBS study was designed primarily to examine changes in cognitive function with endocrine therapy, and thus women older than 65 years were excluded so as not to confound age-related cognitive changes. Initial MBS eligibility criteria included women with diagnosis of stage 0–IIIA breast cancer who had completed their primary cancer treatment within the past 3 months, but had not yet started endocrine therapy. Women could have no major immune-related conditions such as an autoimmune disease, and no evidence of uncontrolled depressive symptoms or neurological conditions. Serial evaluation across numerous domains of biological parameters, cognitive functioning, quality of life, and behavioral symptoms were conducted, and details of patient recruitment from tumor registry listings and direct referral from nearby hospitals and medical oncology practices located near the University of California, Los Angeles, are described in our previous publications.^25,33,42–44^ The initial study included three in-person visits, 6 months apart, during the year after primary treatment ended. MBS participants who completed the 12-month assessment were approached to be in the Long-Term Follow-Up Study, which consisted of annual mailed survey questionnaires and a final in-person visit (TF) that replicated the in-person assessments that had been performed during the first year. Initial study enrollment extended over more than 3 years, resulting in a TF visit that varied from 3–6 years from initial breast cancer treatment. Although 134 completed the TF questionnaire, only 94 attended the in-person visit to provide a fasting morning blood specimen. No differences in medical or demographic data were found among those who provided a blood specimen compared to those who did not at the time of the TF visit. The women seen at the TF visit had remained disease free, with no recurrence of breast cancer. Investigators completing the biological assays were blind to treatment exposure of participants.

### WBC DNA damage

Whole blood samples collected in EDTA tubes were combined with a freezing medium (RPMI + 20% DMSO) and stored at −80 °C until further analyses. DNA damage was determined using the comet assay as reported in Singh et al.^45^ with minor modifications. The comet assay is a single cell gel electrophoresis assay that assessed the extent of DNA damage in nucleated WBC. Cells were thawed and suspended in PBS buffer (Sigma-Aldrich, St. Louis, MO.) which was then added to a 1% low melting agarose (pre-warmed at 37 °C) (LMA, Cat. No. 15517-022, Invitrogen, Life Technologies, Carlsbad, CA). This cell suspension was then placed in rings made in a normal melt agarose gel (BP-160-500, Fisher Scientific, Fair Lawn, NJ) previously poured on hydrophilic side of Gel Bond Film (GelBond® Film, Cat. No. 53734, Cambrex Bio Science Inc., Rockland, ME). Samples were run in triplicate and included positive (normal cells treated with 0.1 M H_2_O_2_) and negative controls (normal cells without treatment). Gel with samples loaded was then treated with a lysis buffer (100 mM Na_2_EDTA pH 10, 2.5 M NaCl, 1% Triton X-100, 10 mM Tris, 10% DMSO) at 4 °C for 1 h. Gel was then washed 3× with neutralizing buffer (0.4 M Tris, pH 7.5), and then placed in the electrophoresis chamber with alkaline buffer (0.3 M NaOH, 1 mM Na_2_EDTA, pH > 13) for 20 min at 4 °C. Electrophoresis was performed in the dark for 45 min, 25 V, and 300 mA at 4 °C, the gel was washed with a neutralizing buffer (0.4 M Tris, pH 7.5). Samples were stained with SYBR Gold (Molecular Probes, Eugene, OR) for visualization of the DNA. Olympus BX51 fluorescent microscope and DP72 camera attached to a FITC filter (Olympus, Cypress, CA) was used to capture images of 100 or more comets containing nuclei. Images were then imported into CASP software (CASP, Wroclaw, Poland) for determination of tail size (length, intensity, area) and head size (radius, intensity, area), and calculations of percentage of DNA in the head and tail. The extent of DNA damage is reported using data from approximately 100 comets per sample using Olive Tail Moment (OTM) values, derived from the percentage of DNA in the tail × distance between the center of the tail and the center of the head. Distribution of DNA damage was not normal, with analyses showing high kurtosis (8.8), resulting in uneven representation of low scores. We created a top quartile cutoff to characterize higher DNA damage (range = 0.8–4.7 OTM) and compare this to the remaining sample with an average low DNA damage scores (range = 0.01–0.79 OTM).

### PBMC telomerase activity

To determine telomerase activity, the telomere repeat amplification protocol (TRAP) was performed as previously described with minor modification.^46,47^ Standard density centrifugation of heparinized whole blood was used to isolate PBMC. Cells were suspended in CHAPS lysis buffer at a concentration of 5000 PBMC per microliter, lysed on ice for 30 min, and then spun at 14,500 rpm at 4° for 20 min. Supernatant was frozen at −80 °C until further analysis. For the assay, thawed telomerase extract equivalent to 15,000 PBMC was mixed with dH2O, 10× TRAP reaction buffer, dNTPs, Cy5-labeled TS primer (5′-ATTCGGTCGACGAGACTT-3′), ACX primer (5′-GCGCGGCTTACCCTTACCCTTACCCTAACC-3′), TSNT (5′-AATCCGTCGAGCAGAGTTAAAAGGCCGAGAAGCGAT-3′), NT primer (5′-ATCGCTTCTCGGCCTTTT-3′), and Platinum Taq for a total volume of 45 µL per reaction. PCR was run as follows, step1: 30 °C for 30 min (1×), step 2: 94 °C for 2 min (1×), then step 3: 94 °C for 30 s, 60 °C for 30 s, 72 °C for 60 s (repeat 28×), and step 4: 72 °C for 60 s. Samples are then frozen at −20 °C before being run in a 12.5% acrylamide gel, which is set up as follows: two negative controls (heat inactivated cell extract per sample and a CHAPS only well containing no cell extract), two positive controls (a telomerase product quantification standard, TSR8, and cell extract with known high telomerase activity), and product generated from sample extracts. As extracts from whole blood may contain PCR inhibitors,^47^ TSNT serves as an internal control. Gels were scanned using a GE Healthcare Typhoon 9410 Variable Mode Imager. Calculation of TPG per 10,000 cells is performed using ImageQuant and calculated as follows: (product generated from the sample)−(heat-treated lane)/(TSNT internal control × 100). This is then divided by (TSR8−CHAPS)/TSNT of TSR8. Values are calculated as total TPG per 10,000 cells, and then transformed to deciles to reduce skew and kurtosis in the distribution of values.

### PBMC telomere length

Detailed methods for the assessment of PBMC telomere length have been reported previously.^48,49^ Briefly, telomere length was determined using standard quantitative polymerase chain reaction methods originally developed by Cawthon.^50^ DNA is extracted from PBMC isolated from heparinized whole blood, using DNeasy Blood and Tissue kits (Qiagen). Samples are run in triplicate on two plates, one for the telomere (TEL) DNA repeats (T) and one for HGB single copy gene (S). Standard curves are generated on each plate to confirm optimal PCR efficiency is obtained, 90–105% and to control for plate-to-plate variations. Inter-assay and intra-assay CV’s were below 10%. Values for PBMC telomere length are expressed as a ratio of TEL to HGB as T/S, which reflects the estimated concentration of the telomere DNA repeat divided by the single copy gene.

### Circulating inflammatory markers: soluble TNF receptor II (sTNF-RII), interleukin(IL)-6, and CRP

Consistent with previous MBS reports,^25,33^ blood samples were collected at the TF study visit by venipuncture into EDTA tubes, chilled, and centrifuged for the collection of plasma. Aliquots of plasma were then stored at −80 °C until batch testing could be performed on all TF samples. Soluble tumor necrosis factor (TNF) receptor type II (sTNF-RII) and Interleukin(IL)-6 were assessed using regular and high-sensitivity enzyme-linked immunosorbent assays (ELISAs; R&D Systems, Minneapolis, MN) per manufacturer’s protocol; lower limits of detection were 234 and 0.2 pg/mL, respectively. CRP levels were determined by a high-sensitivity ELISA (Immundiagnostik, ALPCO Immunoassays, Salem, NH) according to the manufacturer’s protocol, but with an extended standard curve to a lower limit of detection of 0.2 mg/L. All samples were run in duplicate with an average intra-assay precision of less than 5%; inter-assay precision for sTNF-RII, IL-6, and CRP was 4, 12, and 6%, respectively.

### Statistical analyses

Statistical analyses were performed using IBM SPSS for Windows version 23. Variables were assessed for normal distribution and transformed as described above. Linear and logistic regression analyses were run, entering age, BMI, race, and time since treatment as covariates on the first step, and next entering on step 2 treatment type compared to surgery alone on DNA damage, telomerase activity, and telomere length (T/S). First, to determine the overall impact of exposure to either type of treatment, we examined the effect of treatment with chemotherapy and/or with radiation therapy exposure compared to surgery alone. In secondary analyses, we then examined whether chemotherapy and radiation therapy exposure may have distinct effects on cellular aging, we tested whether each of these treatment exposures were uniquely related to outcomes individually compared to surgery alone (see Supplemental Files) using separate regression analyses for each treatment type. Further regression analyses were run to test the relationship of DNA damage, telomerase activity, and telomere length with circulating markers of inflammation (sTNF-RII, IL-6, and CRP). In Table 2 we report adjusted odds ratio (OR) with confidence interval (CI) and unstandardized regression coefficients (*β*) with SE to provide effect size estimates given multiple comparisons.

### Data availability

The data that support the findings of this study are available from the corresponding author upon reasonable request.

## Electronic supplementary material

Supplemental Material
