# Supplementary material for: Biomarkers of aging associated with past treatments in breast cancer survivors
Source: NPJ Breast Cancer. 2017 Dec 12;3:50. doi: 10.1038/s41523-017-0050-6 (PMC5727230; doi:10.1038/s41523-017-0050-6)
Supplement: Supplementary file 1 — Supplemental Material [file 41523_2017_50_MOESM1_ESM.docx]

**Biomarkers of Aging Associated with Past Treatments in Breast Cancer Survivors**

**Supplemental Materials**

**Secondary Analyses**

Secondary analyses tested whether radiation alone, chemotherapy alone, or both chemotherapy and radiation combined would be related to biomarkers compared to surgery alone. Supplemental Table 1 displays the demographic and treatment characteristics within each of these groups.

*Radiation Alone Exposure*

Prior exposure to radiation therapy alone (without receiving chemotherapy) was also examined. In adjusted models (Supplementary Table 2), women who had been exposed to radiation therapy only (n=15), without receiving any chemotherapy, were more likely to be in the high DNA damage category compared to those who had received surgery alone (p=0.03), and were also more likely to have lower telomerase activity (p=0.004). There was no relationship of radiation alone on telomere length.

*Chemotherapy Alone Exposure*

Prior chemotherapy exposure alone (without radiation therapy) on markers of cellular aging at TF was examined. In models adjusting for age, BMI, race, and years from last treatment (See Supplementary Table 2), women who had been exposed to chemotherapy alone were not significantly more likely to be in the high DNA damage category compared to those who had surgery alone (P=0.13). Telomerase enzymatic activity in PBMC did not differ significantly in those who had been exposed to chemotherapy alone compared to surgery alone (P=0.21). There were no differences in PBMC telomere length by chemotherapy alone exposure status.

*Both Chemotherapy and Radiation*

We then examined the effect of prior chemotherapy and radiation exposure combined compared to those receiving surgery alone. Women who had been exposed to both chemotherapy and radiation were significantly more likely to have high levels of DNA damage compared to women who received surgery alone (P=0.03). Effects of both chemotherapy and radiation on telomerase and telomere length were not significant (See Supplementary Table 2).

*Chemotherapeutic Regimen*

All patients received a taxane as part of their adjuvant chemotherapy (usually combined with cyclophosphamide or carboplatin). A minority of the patients received an anthracycline as well (n=10), with the remainder not having this exposure (n=41). High DNA damage vs. low DNA damage was slightly more common in those who received an anthracycline (30% high DNA damage group) in comparison to those who did not receive an anthracycline (22% high DNA damage group); however these differences were not statistically significant (P=0.59). Exposure to anthracycline was not associated with PBMC telomere length nor telomerase activity.

|  | Total Sample | Surgery  Alone | Chemotherapy  Alone | Radiation Therapy Alone | Both Chemo & Radiation | P Value |
| --- | --- | --- | --- | --- | --- | --- |
|  | N=94 | N=15 | N=11 | N=28 | N=40 |  |
| Age, mean (SD) | 56.5 (8.1) | 58.1 (5.4) | 50.4 (6.5) | 59.0 (7.8) | 55.8 (8.7) | .02 |
| Body Mass Index (BMI), mean (SD) | 25.7 (5.1) | 26.0 (5.6) | 24.7 (4.9) | 25.7 (5.2) | 25.8 (5.0) | .92 |
| Years since diagnosis, mean (SD) | 4.8 (0.7) | 4.7 (.60) | 4.8 (.96) | 4.8 (.56) | 4.9 (.68) | .89 |
| Years since last treatment, mean (SD) | 4.4 (0.6) | 4.6 (.57) | 4.4 (.84) | 4.5 (.55) | 4.3 (.44) | .29 |
| Race, % White | 80% | 86.7% | 72.7% | 85.7% | 75% | .58 |
| Marital Status, % Married | 62.8% | 53.3% | 64% | 67.9% | 62.5% | .83 |
| Education  Post college  College  No college degree | 50%  31%  19% | 53.3%  33.3%  13.3% | 45.5%  45.5%  9.1% | 57.1%  17.9%  25% | 45%  35%  20% | .61 |
| Employment status  Full or part-time  Not employed | 73%  27% | 86.7%  13.3% | 63.6%  36.4% | 60.7%  39.3% | 65%  35% | .35 |
| Annual household income  >=$100,000  <$100,000 | 59.6%  40.4% | 66.7%  33.3% | 54.5%  45.5% | 71.4%  28.6% | 50%  50% | .31 |
| Post-menopausal | 80.9% | 80% | 72.7% | 82.4% | 80% | .82 |
| Past hormone therapy (HT) | 33.3% | 28.6% | 10% | 50% | 30.8% | .09 |
| Surgery  Mastectomy  Lumpectomy | 34%  66% | 80%  20% | 100%  0% | 0%  100% | 22.5%  77.5% | .000 |
| Stage at diagnosis  Stage 0  Stage I  Stage II  Stage III | 16%  46%  31%  7% | 53.3%  46.7%  0%  0% | 0%  36.4%  63.6%  0% | 25%  64.3%  10.7%  0% | 0%  35%  47.5%  17.5% | .000 |
| Endocrine Therapy | 72% | 40% | 0% | 85.7% | 72.5% | .01 |
| DNA Damage (% High) | 24.5% | 7% | 18% | 36% | 25% | .19 |
| WBC Telomerase TPG per 10,000 cells, mean (SD) | 22.7(19.4) | 30.3 (20.2) | 14.6 (8.9) | 14.1 (11.2) | 28.3 (22.9) | .02 |
| PBMC Telomere Length, Mean (SD) | .72 (.27) | .71 (.37) | .77 (.29) | .68 (.25) | .76 (.31) | .39 |
| Plasma Cytokines, mean (SD)  IL-6, pg/mL  sTNF-RII, pg/mL  CRP, mg/L | 1.3 (.99)  2228.4 (530.9)  2.4 (2.7) | 1.28 (.87)  2093.6 (459.7)  2.26 (2.7) | 1.45 (1.6)  2255.7 (597.2)  1.85 (1.2) | 1.20 (.91)  2323.8 (599.78)  2.53 (2.2) | 1.26 (.92)  2200.6 (488.74)  2.57 (3.3) | .92  .59  .88 |

**Supplemental Table 1. Participant Medical and Demographic Characteristics Overall and Stratified by Treatment Group**

| **Supplemental Table 2. Multivariate analyses examining treatment exposure subtypes predicting biomarkers of aging at final visit (TF) adjusting for age, race, BMI, and years from treatment** | | | | | | | | | | | |
| --- | --- | --- | --- | --- | --- | --- | --- | --- | --- | --- | --- |
|  | WBC DNA Damage  (High Damage vs. Low) | | | | | PBMC Telomerase  (Deciles) | | | PBMC Telomere Length  (T/S) | | |
| *Treatment Exposure* | *β*(SE)^1^ | | OR(95% CI)^2^ | p-value | | *β*(SE) | Beta^3^ | p-value | *β*(SE) | Beta | p-value |
| Radiation Therapy Alone | | 3.18(1.46) | 23.93(1.36, 420.95) | | .03 | -2.75(.88) | -.45 | .004 | -.03(.09) | -.05 | .73 |
| Chemotherapy Alone | | 2.87(1.9) | 17.64(0.42, 750.15) | | .13 | -2.15(1.6) | -.38 | .21 | .23(.17) | .34 | .20 |
| Both Chemotherapy & Radiation | | 6.48(3.05) | 4.51(1.65, 25,980) | | .03 | -1.28(.99) | -.20 | .20 | .09(.10) | .14 | .37 |
|  | |  |  | |  |  |  |  |  |  |  |

1: Standard coefficient (*β)* and standard error (SE)

2: Odds Ratio (OR) and 95% confidence interval (95% CI)

3: Unstandardized regression coefficient (Beta)
